# Paediatric Sleep-Disordered Breathing: Pharyngeal Airway and Lymphoid Tissues in Risk Assessment

**DOI:** 10.3390/jcm15134991

**Published:** 2026-06-26

**Authors:** Sandra Yi Cheng Chee, Lynn Huiting Koh, Kelvin Weng Chiong Foong, Clement Wei Ming Lai, Yu Fan Sim, Mimi Yow

**Affiliations:** 1 Department of Orthodontics, Faculty of Dentistry, National University of Singapore, 9 Lower Kent Ridge Rd, Singapore 119085, Singapore; 2 Department of Otolaryngology, KK Women’s and Children’s Hospital, 100 Bukit Timah Road, Singapore 229899, Singapore; lynn.koh.h.t@singhealth.com.sg; 3 Faculty of Dentistry, National University of Singapore, 9 Lower Kent Ridge Rd, Singapore 119085, Singapore; denfwc@nus.edu.sg; 4 Biostatistics Unit, Faculty of Dentistry, National University of Singapore, 9 Lower Kent Ridge Rd, Singapore 119085, Singapore; clelwm@nus.edu.sg (C.W.M.L.); densyf@nus.edu.sg (Y.F.S.); 5 Department of Orthodontics, National Dental Centre Singapore, 5 Second Hospital Ave, Singapore 168938, Singapore; mimi.yow@singhealth.com.sg

**Keywords:** orthodontics, sleep-disordered breathing, obstructive sleep apnea, craniofacial abnormalities, upper airway, cephalometry

## Abstract

**Background/Objectives:** Upper airway constriction and craniofacial structural variation are recognised risk factors for paediatric sleep-disordered breathing (SDB). Population-specific normative cephalometric reference data are lacking, and are needed to characterise these features in paediatric orthodontic patients, especially in Asian populations. This study examined upper airway structure and lymphoid tissue hypertrophy in a large paediatric orthodontic population. The aims of the study were to investigate upper airway differences across skeletal patterns, age, gender, and ancestry groups, establish pharyngeal airway, skeletal, dental, and soft tissue cephalometric dimensions, and determine adenotonsillar hypertrophy prevalence in a large paediatric orthodontic population in Singapore. **Methods:** Lateral cephalograms of children aged 7–11 years were obtained from a national dental centre, and a retrospective analysis was performed. Standardised cephalometric measurements were used to assess airway, skeletal, dental, and soft tissue parameters, with comparisons across demographic and skeletal groups. **Results:** A total of 404 children (203 boys, 201 girls; aged 7.04–10.99 years) were included in the final analysis. Thirteen airway variables differed significantly by gender and age, six by antero-posterior, and four by vertical skeletal pattern. One variable (AH-CV) differed between Chinese and non-Chinese children. A form of lymphoid tissue hypertrophy (Ad/Np ≥ 0.5 and/or Tn/Op ≥ 0.5) was present in 92.3% of subjects, comprising combined adenotonsillar hypertrophy (49.5%), isolated tonsillar hypertrophy (36.6%), and isolated adenoid hypertrophy (6.2%). **Conclusions:** Cephalometric norms for upper airway, skeletal, dental, and soft tissue structures in a 7–11-year-old orthodontic population in Singapore were reported. Adenotonsillar hypertrophy was present in nearly half of the subjects, while isolated tonsillar hypertrophy affected about one-third. Patients who were younger, male, Chinese, Class I, Class II, and had increased mandibular plane angles displayed cephalometric features associated with anatomical risk indicators for SDB. These population-specific cephalometric reference data provide a benchmark for contextualising upper airway and craniofacial measurements in paediatric orthodontic patients, supporting the potential utility of cephalometric assessment to identify children who may benefit from referral for comprehensive SDB evaluation.

## 1. Introduction

Sleep-disordered breathing (SDB) in children represents a significant clinical concern, with implications for cardiovascular health, neurocognitive development, somatic growth delay and/or failure, and overall paediatric wellbeing [1]. SDB encompasses a spectrum of nocturnal breathing disorders characterised by increased upper airway resistance and partial or complete upper airway obstruction [2]. For clinicians treating young children, understanding the relationship between upper airway anatomy and SDB is crucial, as early identification and intervention can prevent serious long-term sequelae. Research has found associations between upper airway calibre and craniofacial and overall development in the growing child [3,4,5,6]. While genetic factors do play a primary role in craniofacial development, current evidence suggests a complex cyclical relationship between adenotonsillar hypertrophy, oral breathing patterns, and craniofacial development, which predisposes a subset of children to SDB and related morbidities from a young age [7].

The prevalence of paediatric OSA ranges globally from 0.7 to 5.7% [3,8,9,10,11,12,13,14,15]. In Singapore, the estimated prevalence of obstructive sleep apnoea (OSA) syndrome was found to be 5.7% and 13.3% in obese and morbidly obese school children, respectively, with adenotonsillar hypertrophy also identified as a primary risk factor [16]. One local centre reported the prevalence of OSA in overweight children to be much higher; approximately 55–64% [17]. The intersection with mental health is also striking, with 62.2% of children attending psychiatric services and 77.3% of children diagnosed with attention-deficient/hyperactivity disorder (ADHD) in Singapore experiencing sleep disturbances [18]. Importantly, Chng et al. [19] found significant improvements in quality of life after treatment of OSA in Singaporean children, highlighting the importance of identifying children at risk of SDB for early intervention. A recently published consensus statement [20] made strong recommendations for applying a lower threshold of screening and evaluation for children with craniofacial abnormalities that increase the risk of OSA, which forms a significant proportion of children seeking orthodontic treatment.

Aside from adenotonsillar hypertrophy and obesity, certain craniofacial anomalies have also been identified as risk factors for paediatric OSA [21]. Adenoids, tonsils, and other soft and hard craniofacial structures can be visualised on lateral cephalograms. Given that lateral cephalograms are routinely taken for orthodontic treatment planning, these radiographs present an opportunity for SDB risk screening in the paediatric orthodontic population [22,23,24]. As part of a comprehensive SDB risk assessment protocol involving history reviews, questionnaires, and clinical and radiographic evaluations [25], cephalometric analysis of lateral radiographs can serve as a screening adjunct for orthodontists and paediatric dentists to identify children with anatomical risk indicators who may benefit from further clinical evaluation and referral.

Normative cephalometric airway values for Southeast Asian children aged 7–11 years are lacking. Furthermore, no large-scale dataset integrating skeletal classification, vertical pattern, ancestry, and upper airway dimensions exists for this paediatric orthodontic population in Singapore. Hence, this study is significant as it addresses a critical gap by establishing the first set of cephalometric upper airway reference values for Singaporean children. Our objectives were to (1) establish upper airway, skeletal, dental, and soft tissue cephalometric measurements in this population, (2) determine adenotonsillar hypertrophy prevalence in this population, and (3) investigate airway measurement differences across skeletal patterns, age, gender, and ancestry groups. We hypothesised that various craniofacial and upper airway cephalometric measurements, as well as lymphoid tissue hypertrophy prevalence rates would differ significantly across certain subgroups. These data provide population-specific upper airway and craniofacial cephalometric reference values. This supports the utility of incorporating upper airway cephalometric assessment into routine orthodontic screening to aid clinicians in identifying patients who may then benefit from referral for formal SDB evaluation. We would like to emphasise that this study does not include polysomnography or clinical sleep assessment; all findings represent cephalometric anatomical associations, and are not intended to inform formal clinical diagnoses of SDB.

## 2. Materials and Methods

The study involved analysis of third-party de-identified clinical radiographic records obtained in the course of routine patient care, with no direct patient contact or intervention. Hence, the requirement for patient informed consent and Centralised Institutional Review application was waived by the SingHealth Centralised Institutional Review Board. The study was approved by, and radiographic data were obtained from, the National Dental Centre Singapore (project number: 380/2022). This retrospective study complied with STROBE guidelines [26]. Consecutive lateral cephalograms were analysed to obtain upper airway, skeletal, dental, and soft tissue cephalometric measurements.

### 2.1. Materials

The materials used were lateral cephalograms of 7- to 11-year-olds, obtained from the digital radiograph database of the National Dental Centre Singapore (NDCS), between 1 February 2018 to 31 October 2023. The lateral cephalograms utilised were indicated as part of routine pre-treatment investigations for orthodontic diagnosis of patients seeking orthodontic treatment at the NDCS.

Inclusion criteria were lateral cephalograms (1) of subjects between 7 and 11 years of age at the time of radiographic exposure, which (2) had adequate clarity of craniofacial and upper airway structures. Exclusion criteria included lateral cephalograms (1) with displaced tongue and soft palate due to swallowing, (2) patients who had previous or were undergoing orthodontic treatment, and (3) patients who had histories of cleft and craniofacial anomalies and/or syndromes. Lateral cephalograms with displaced tongue and soft palate were identified based on characteristic radiographic features of mid-swallow positioning, including elevation of the tongue dorsum approximating the soft palate and obliteration of the nasopharyngeal airway silhouette. This exclusion was applied visually by a single trained operator.

### 2.2. Methods

#### 2.2.1. Radiographic Technique

The radiographs were taken by radiographers using ORTHOPANTOMOGRAPH™ OP 3D Pro (KaVo Dental, Biberach, Germany). Subjects were in upright positions, and were aligned for natural head posture using the machine’s ear rods, nasion support bar, and Frankfurt horizontal light. They were instructed to bite on their back teeth and relax their lips. The exposure used was 85 kV, 10.0 mA, with 13 s at 44 mGycm^2^. Radiographs were archived digitally.

#### 2.2.2. Cephalometric Analysis

Demographic data collected included age at time of radiographic exposure, gender, and ancestry.

**Figure 1:**
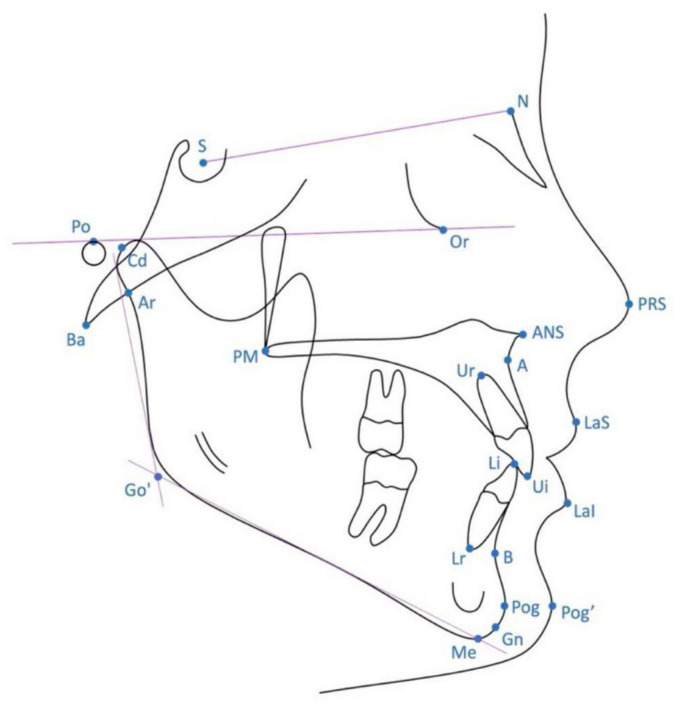
Skeletal, dental, and soft tissue cephalometric landmarks. Definitions of labelled points are provided in the Supplementary Materials.

**Figure 2:**
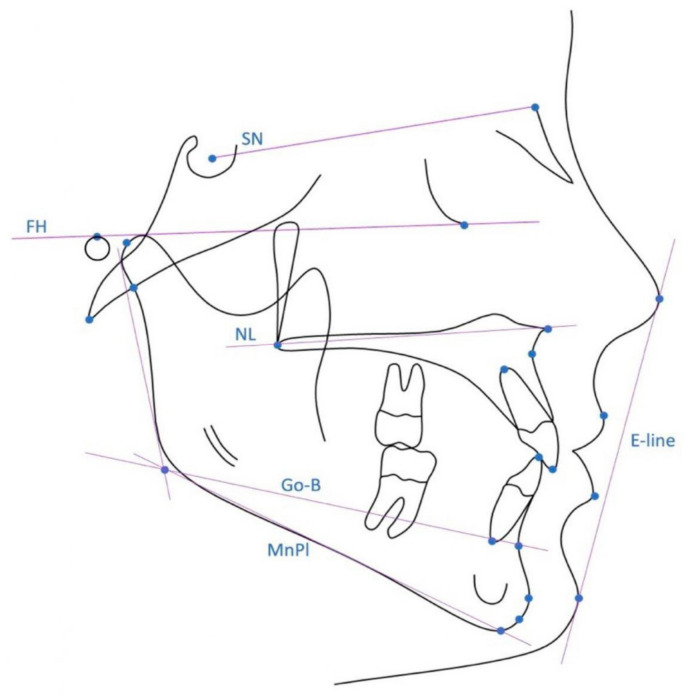
Skeletal, dental, and soft tissue cephalometric reference planes. Definitions of labelled points and planes are provided in the Supplementary Materials.

**Figure 3:**
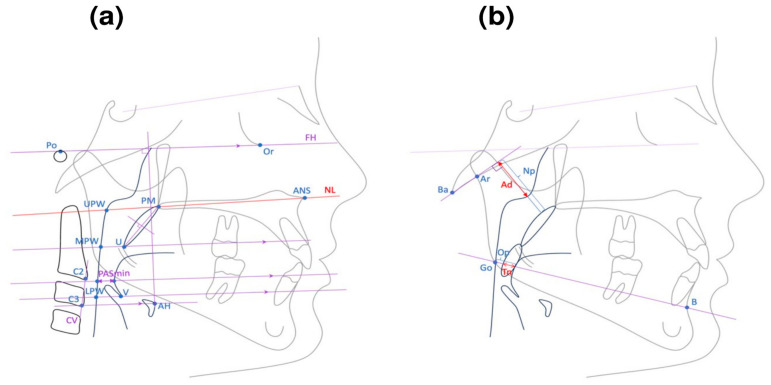
(**a**) Cephalometric landmarks, reference planes, and measurements of the upper posterior airway space; (**b**) cephalometric landmarks, reference planes, and measurements of the adenoids and tonsils and associated airway depths. Definitions of labelled points and planes are provided in the Supplementary Materials, and measurements utilising these points and planes are listed in Table 1.

Cephalometric upper airway, dental, skeletal, and soft tissue landmarks and reference planes are shown in Figure 1, Figure 2 and Figure 3, with their detailed definitions provided in the Supplementary Materials. Cephalometric measurements (Table 1) of upper airway variables included fourteen linear variables and one angular variable (two variables for adenoids, two variables for tonsils, six variables for upper airway depth, three variables for the soft palate, and two variables for the position of the hyoid bone). These variables were selected based on the methodologies established by Gu et al. [27], Baroni et al. [28], and Huang et al. [29] to allow for direct comparisons and to encourage standardisations against their and other authors’ findings in similar populations and/or studies. Skeletal, dental, and soft tissue variables (Table 1) included six linear and ten angular (seven skeletal, seven dental, and two soft tissue) conventional cephalometric variables. Cephalometric analysis was completed using Audaxceph^®^ software version 6.7.10 (Audax, d.o.o., Ljubljana, Slovenia). All cephalometric landmark identification and measurements were performed ex novo for this study by a single trained operator, who was a final (third) year orthodontics resident with 3 years of experience in cephalometric analysis. The dataset was checked twice in its entirety for accuracy in landmark identification and calibration.

#### 2.2.3. Error of the Method

To determine intra-examiner reliability, 35 lateral cephalograms were randomly selected for re-measurement two months after initial measurements. The mean ICC of this project was 0.95 (SD: 0.18), indicating excellent intra-examiner reliability.

#### 2.2.4. Statistical Analysis

Mean (SD) values of all cephalometric variables for subjects of different age and gender groups were calculated. The sample size to determine the upper posterior airway space in each of the targeted groups was calculated. Based on the findings from Masoud and Alwadei’s [30] study, the standard deviation (SD) for airway spaces ranges from 2.09 to 3.50. A total of 99 samples per group were required to estimate the upper posterior airway space, with a margin of error of 0.7 and 95% confidence interval.

Differences between groups based on age and gender, antero-posterior and vertical skeletal classifications, and ancestry were investigated. Three groups of antero-posterior skeletal patterns (Class I, II, III) were defined by ANB values of 0.9–5.9°, >5.9°, and <0.9°, respectively. Similarly, three groups of vertical skeletal patterns (high-, average- and low- angle groups) were defined by MMPA values of >31.4°, 21.1–31.4°, and <21.1°, respectively [31]. Due to the limited number of non-Chinese (Malay, Indian, and Caucasian) subjects within the sample, these subjects were combined into a single group for analysis. Patients were also categorised into four different groups for adenoid and/or tonsillar size based on Ad/Np and Tn/Op ratios, where an Ad/Np ratio > 0.5 was considered to constitute adenoid hypertrophy, a Tn/Op ratio > 0.5 was considered to constitute tonsillar hypertrophy, and Ad/Np and Tn/Op ratios both >0.5 were considered to constitute adenotonsillar hypertrophy [28,29].

The sample size to determine differences between age and gender groups was calculated. A sample size of 400 was required to achieve a statistical power of 80% at an overall significance level of 5%, effect size of 0.5, and an allocation ratio of 1:1. ANOVA or *t*-tests were conducted to determine if there were statistically significant differences between groups. Once the results of the ANOVA were statistically significant, post hoc analysis using Bonferroni correction was done to determine which pairs of the groups had statistically significant differences. All statistical analyses were performed using R version 4.3.1 (The R Foundation for Statistical Computing, Vienna, Austria).

#### 2.2.5. Data Availability

De-identified data can be made available via request to any of the corresponding authors of the current study.

## 3. Results

### 3.1. Participant Flow

**Figure 4:**
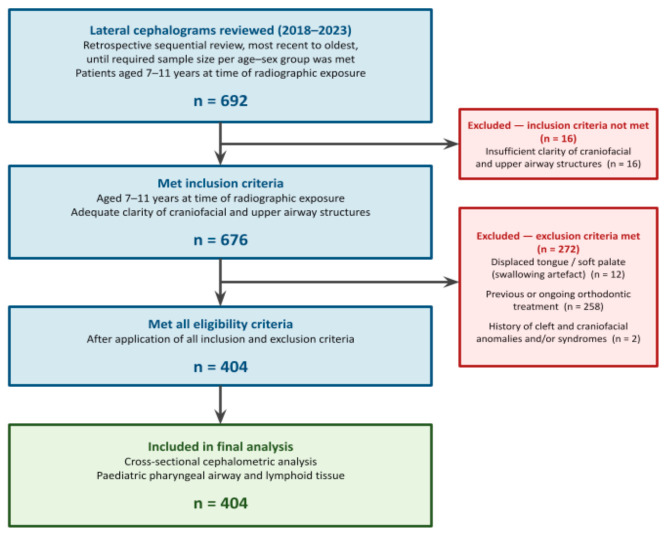
STROBE participant flow diagram. Lateral cephalograms (2018–2023) were reviewed sequentially from most recent to oldest until the required sample size per age–gender group was met. Of 692 cephalograms screened, 288 were excluded: 16 for insufficient craniofacial/upper airway clarity; 12 for swallowing artefacts; 258 for previous/current orthodontic treatment; and 2 for craniofacial anomalies/syndromes.

Lateral cephalograms were retrieved from the digital radiographic database of the NDCS. The following flowchart (Figure 4) describes the selection process of the cephalograms included in final analysis.

### 3.2. Demographic Characteristics of the Subjects

The study included a total of 404 subjects. Demographic characteristics of the subjects are reported in Table 2.

### 3.3. Craniofacial Non-Airway Measurements

All cephalometric measurements categorised by different groupings and comparisons between groups are provided in the Supplementary Materials.

**Figure 5:**
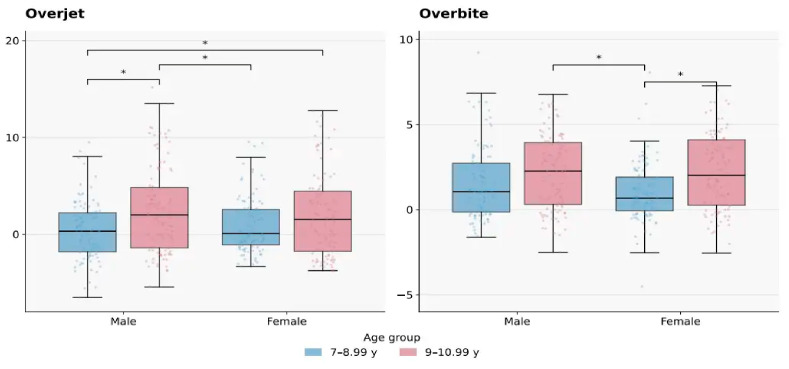
Overjet and overbite distributions across age and gender groups. Box plots showing the median (interquartile range) and individual data points (jittered) for overjet (**left**) and overbite (**right**) in millimetres, stratified by sex and age group (blue: 7–8.99 years; pink: 9–10.99 years). Brackets with asterisks (*) denote statistically significant pairwise differences (one-way ANOVA with Tukey HSD post-hoc; * *p* < 0.05; overall *p* < 0.001 for both measurements).

Mean (SD) values of all cephalometric variables for subjects of different age and gender groups were calculated. There were significant differences between gender and age groups for seven out of sixteen craniofacial non-airway measurements (*p* ≤ 0.032; all measurements presented in the Supplementary Materials), with overjet and overbite measurements reaching the highest significance levels for differences (*p* < 0.001) between groups (Figure 5).

Older boys (9–10.99 y) had the largest mean overjet (2.52 ± 4.48 mm), which was significantly greater than both younger (7–8.99 y) boys (0.47 ± 3.12 mm) and younger girls (1.03 ± 2.93 mm). Older girls (2.00 ± 4.32 mm) did not differ significantly from older boys, but had significantly greater overjet than younger boys. Younger boys and younger girls did not differ significantly from each other.

Younger girls had the smallest mean overbite (1.01 ± 1.75 mm), which was significantly smaller than both older boys (2.19 ± 2.19 mm) and older girls (2.23 ± 2.29 mm). No other pairwise differences were statistically significant; younger boys (1.56 ± 2.15 mm) did not differ significantly from any other group.

### 3.4. Airway Measurements

**Figure 6:**
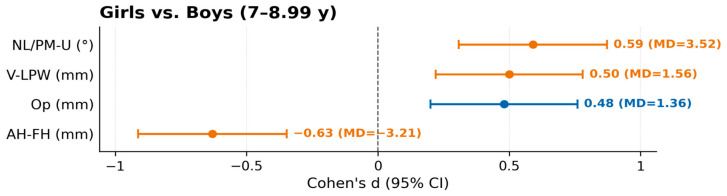
Cohen’s d forest plot: Girls vs. Boys (7–8.99 years; n = 101 per group). Cohen’s d effect sizes (95% CI) for significant upper airway cephalometric differences between girls and boys aged 7–8.99 years. Colour denotes effect size magnitude: blue = small (0.2 ≤ |d| < 0.5); orange = medium (0.5 ≤ |d| < 0.8). MD = mean difference in original units.

**Figure 7:**
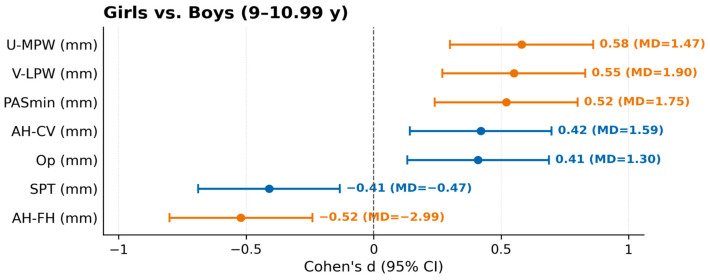
Cohen’s d forest plot: Girls vs. Boys (9–10.99 years; n = 103 boys, n = 100 girls). Cohen’s d effect sizes (95% CI) for significant upper airway cephalometric differences between girls and boys aged 9–10.99 years. Colour denotes effect size magnitude: blue = small; orange = medium.

**Figure 8:**
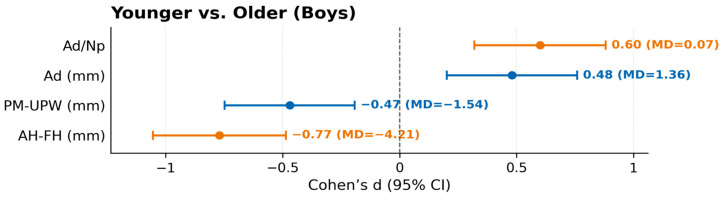
Cohen’s d forest plot: Younger vs. Older boys (7–8.99 years, n = 101; 9–10.99 years, n = 103). Cohen’s d effect sizes (95% CI) for significant upper airway cephalometric differences between younger and older boys. Colour denotes effect size magnitude: blue = small; orange = medium.

**Figure 9:**
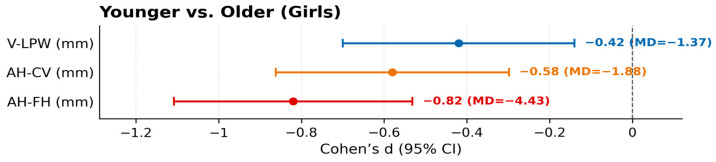
Cohen’s d forest plot: Younger vs. Older girls (7–8.99 years, n = 101; 9–10.99 years, n = 100). Cohen’s d effect sizes (95% CI) for significant upper airway cephalometric differences between younger and older girls. The AH-FH difference represented a large effect size (|d| = 0.82). Colour denotes effect size magnitude: blue = small; orange = medium; red = large.

**Figure 10:**
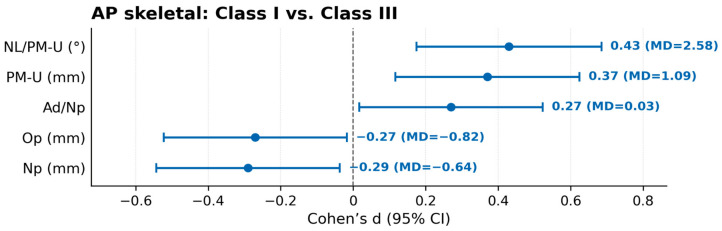
Cohen’s d forest plot: AP skeletal Class I vs. Class III. Cohen’s d effect sizes (95% CI) for significant upper airway cephalometric differences between Class I and Class III subjects. All differences were of small effect size. Colour denotes effect size magnitude: blue = small.

**Figure 11:**
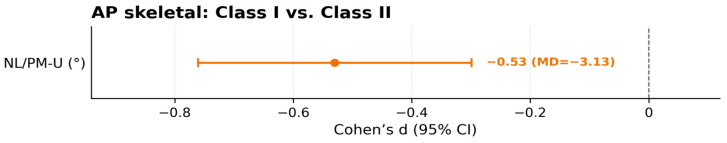
Cohen’s d forest plot: AP skeletal Class I vs. Class II. Cohen’s d effect size (95% CI) for significant upper airway cephalometric differences between Class I and Class II subjects. Colour denotes effect size magnitude: orange = medium.

**Figure 12:**
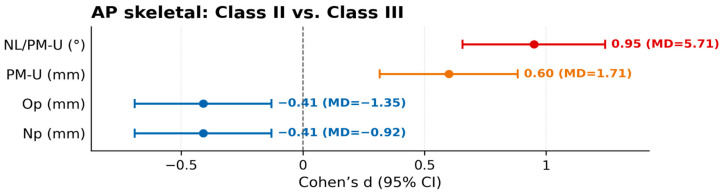
Cohen’s d forest plot: AP skeletal Class II vs. Class III. Cohen’s d effect sizes (95% CI) for significant upper airway cephalometric differences between Class II and Class III subjects. Colour denotes effect size magnitude: blue = small; orange = medium; red = large.

**Figure 13:**
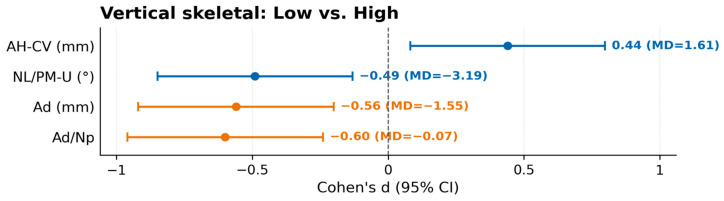
Cohen’s d forest plot: Vertical skeletal Low vs. High angle. Cohen’s d effect sizes (95% CI) for significant upper airway cephalometric differences between low and high vertical skeletal patterns. Colour denotes effect size magnitude: blue = small; orange = medium.

**Figure 14:**
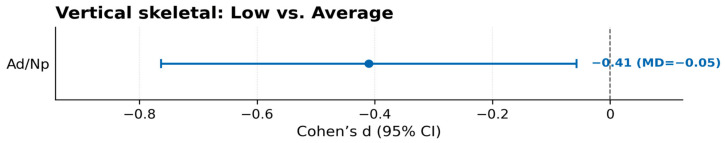
Cohen’s d forest plot: Vertical skeletal Low vs. Average angle. Cohen’s d effect size (95% CI) for the significant upper airway cephalometric difference between low and average vertical skeletal patterns. Colour denotes effect size magnitude: blue = small.

**Figure 15:**
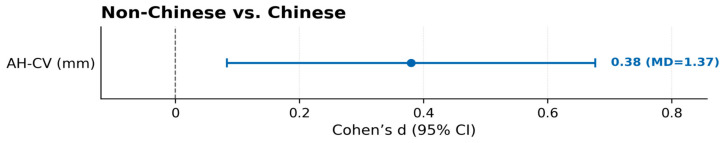
Cohen’s d forest plot: Non-Chinese vs. Chinese. Cohen’s d effect size (95% CI) for the significant upper airway cephalometric difference between non-Chinese and Chinese subjects. Colour denotes effect size magnitude: blue = small.

Differences in airway measurements between various groups were analysed, and forest plots were generated to reflect effect sizes where significant differences were found. Positive Cohen’s d values indicate larger measurements in the comparison group; negative values indicate larger measurements in the reference group. Error bars represent 95% confidence intervals. MD = mean difference in original measurement units. Only variables reaching statistical significance (*p* < 0.05) with |d| ≥ 0.20 are displayed (Figure 6, Figure 7, Figure 8, Figure 9, Figure 10, Figure 11, Figure 12, Figure 13, Figure 14 and Figure 15).

#### 3.4.1. Gender Differences in Airway Measurements

Subjects categorised by age and gender significantly differed in thirteen out of fifteen airway measurements (*p* < 0.005) (Table 3).

Younger (7- to 8.99- year-old) girls had significantly greater oropharyngeal widths at the tonsillar level (*p* = 0.004), soft palate inclinations (*p* < 0.001), hypopharyngeal airway depths (*p* = 0.003), and more superiorly positioned hyoid bones (*p* < 0.001) than younger (7- to 8.99-year-old) boys.

In the older (9- to 10.99-year-old) age group, girls had significantly greater oropharyngeal widths at both the tonsillar (*p* = 0.023) and uvula (*p* < 0.001) levels, minimum airway space measurements (*p* = 0.002), and hypopharyngeal airway depths (*p* < 0.001). They also had thinner soft palates (*p* = 0.026) and more superiorly (*p* = 0.002) and anteriorly (*p* = 0.021) positioned hyoid bones than their male counterparts.

There were more gender differences in airway measurements for the older age group than in the younger age group.

#### 3.4.2. Age Differences in Airway Measurements

Younger (7- to 8.99-year-old) boys had greater adenoid hypertrophy (*p* < 0.001), wider adenoid widths (*p* = 0.005), smaller retropalatal airway depths (*p* = 0.006), and more superiorly positioned hyoid bones (*p* < 0.001) than older (9- to 10.99-year-old) boys.

Younger girls had smaller hypopharyngeal airway depths (*p* = 0.02), and more superiorly (*p* < 0.001) and posteriorly (*p* < 0.001) positioned hyoid bones than older girls.

There were a greater number of differences found between genders compared to between age groups.

#### 3.4.3. Differences in Airway Measurements Between Subjects of Different Antero-Posterior Skeletal Patterns

Class I, II, and III subjects comprised 55.0%, 11.1%, and 33.9% of the sample, respectively. Subjects of different antero-posterior skeletal classifications differed in eleven out of sixteen non-airway cephalometric measurements (*p* < 0.001), and six out of fifteen airway measurements (*p* < 0.05).

Class III subjects had significantly greater nasopharyngeal width at the level of the adenoids (23.68 ± 2.34 mm, *p* = 0.009), oropharyngeal width at the level of the tonsils (12.26 ± 3.46 mm, *p* = 0.010), smaller soft palate inclinations (127.19 ± 6.14°, *p* < 0.001), and shorter soft palates (25.76 ± 2.61 mm, *p* < 0.001) compared to Class I and II subjects. Class III subjects demonstrated significantly smaller soft palate inclinations (*p* < 0.001), soft palate lengths (*p* = 0.001), less adenoid hypertrophy (*p* = 0.037), and larger oropharyngeal widths at the level of the tonsils (*p* = 0.045), and nasopharyngeal widths at the level of the adenoids (*p* = 0.029) compared to Class I subjects (Figure 10).

Class II subjects had the narrowest nasopharyngeal widths at the level of the adenoids (22.76 ± 2.08 mm), oropharyngeal widths at the level of the tonsils (10.91 ± 2.53 mm) and uvula (9.51 ± 2.42 mm), hypopharyngeal (12.51 ± 2.96 mm), and minimum (9.10 ± 2.99 mm) airway depths, and the most inferiorly (71.52 ± 6.38 mm) and posteriorly (28.54 ± 3.63 mm) positioned hyoid bones among the groups, although most of these differences did not reach statistical significance. Class II subjects demonstrated significantly larger soft palate inclinations (*p* = 0.004) compared to Class I subjects (Figure 11). Class II subjects demonstrated markedly larger soft palate inclinations (d = 0.95, *p* < 0.001) and lengths (*p* = 0.009), and smaller oropharyngeal widths at the level of the tonsils (*p* = 0.017) and nasopharyngeal widths at the level of the adenoids (*p* = 0.043) compared to Class III subjects (Figure 12).

For tonsil width, significant difference(s) were found between groups, but there was insufficient power to determine the specific pair(s) of groups that had difference(s) via post hoc analysis.

#### 3.4.4. Differences in Airway Measurements Between Subjects of Different Vertical Skeletal Patterns

Average-, high-, and low-angled patients determined by MMPA comprised 71.8%, 15.1%, and 13.1% of the sample, respectively.

Subjects of different vertical skeletal patterns differed in ten out of sixteen non-airway cephalometric measurements (*p* ≤ 0.002), and in four out of fifteen airway measurements (*p* < 0.05).

Low-angle subjects had more anteriorly positioned hyoid bones (*p* = 0.045), smaller soft palate inclinations (*p* = 0.019), and smaller adenoid widths (*p* = 0.007) than high-angle subjects, and less adenoid hypertrophy than both high- (*p* = 0.003) and average-angle (*p* = 0.005) subjects (Figure 13 and Figure 14).

#### 3.4.5. Differences in Airway Measurements Between Subjects of Different Ancestries

Non-Chinese participants were grouped together due to insufficient non-Chinese subjects for meaningful statistical analysis with separate groupings. This comprised Malay, Indian, Eurasian, and Caucasian ancestries. Non-airway measurements of subjects categorised by ancestry are reported in the Supplementary Materials.

Chinese and non-Chinese subjects differed in nine out of sixteen non-airway cephalometric measurements (*p* ≤ 0.037), and one out of fifteen airway variables (*p* = 0.01).

Non-Chinese subjects had more anteriorly positioned hyoid bones (*p* = 0.011) compared to Chinese subjects (Figure 15).

### 3.5. Lymphoid Tissue Hypertrophy

**Figure 16:**
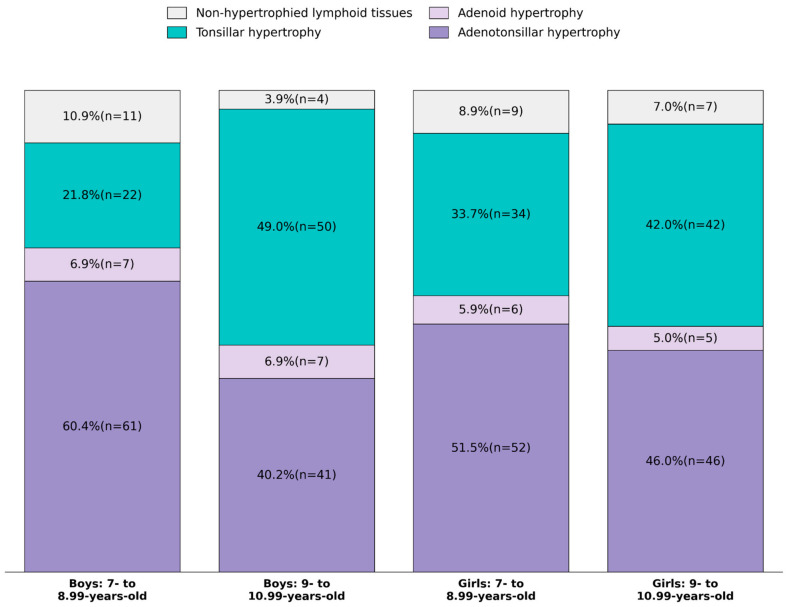
Prevalence of non-hypertrophied lymphoid tissues, tonsillar hypertrophy, adenoid hypertrophy, and adenotonsillar hypertrophy in different age and gender groups.

The prevalence (95% CI) of non-hypertrophied lymphoid tissues, tonsillar hypertrophy, adenoid hypertrophy, and combined adenotonsillar hypertrophy are reported in Table 4. Combined adenotonsillar hypertrophy was the most prevalent in all groups, except in older (9- to 10.99-year-old) boys, where tonsillar hypertrophy was the most prevalent finding (49.0%). Younger (7- to 8.99-year-old) boys were more likely to have either non-hypertrophied lymphoid tissues (10.9%) or combined adenotonsillar hypertrophy (60.4%) when compared to subjects in the other groups (Figure 16).

In the younger age group, girls were more likely to have tonsillar hypertrophy (33.7%) than boys (21.8%), but less likely to have adenoid hypertrophy, combined adenotonsillar hypertrophy, and non-hypertrophied lymphoid tissues. Combined adenotonsillar hypertrophy was the most prevalent finding, followed by tonsillar hypertrophy, non-hypertrophied lymphoid tissues and, lastly, adenoid hypertrophy in both boys and girls.

In the older age group, boys were most likely to have tonsillar hypertrophy (49.0%) and least likely to have non-hypertrophied lymphoid tissues (3.9%), while girls were most likely to have combined adenotonsillar hypertrophy (46.0%) and least likely to have adenoid hypertrophy (5.0%).

## 4. Discussion

This study presents cephalometric measurements for a paediatric orthodontic population in Singapore, using standardised definitions from Gu et al. [27], to facilitate comparisons across studies. A recent investigation of age-matched Chinese children reported similar upper airway measurements, and noted that obesity and adenoid hypertrophy accounted for 40% and 14.7% of the variance in the obstructive apnoea–hypopnea index (OAHI) among 8–10-year-olds [32]. Pharyngeal soft tissues exhibit rapid growth from ages 6–9 and 12–15 years, with quiescent intervals at 9–12 and 15–18 years. After age 9, the soft palate lengthens by approximately 1 mm every three years, while the hyoid bone shifts anteriorly and descends until age 18, influenced by mandibular forward translation and cervical vertebral growth [33,34,35].

Younger male children, and those with Class I or Class II skeletal patterns, were found to have consistently smaller airway depths compared to females, older children, and Class III subjects. Narrow airway dimensions are associated with increased collapse risk via mechanisms described by Poiseuille’s law and the Venturi effect. Constricted airways are associated with turbulent airflow, greater resistance, and higher ventilatory pressures [36,37]. Cai et al. [38] demonstrated that when adenoid hypertrophy obstructs approximately two-thirds of the nasopharynx, airflow becomes turbulent, precipitating pharyngeal collapse, snoring, hypopnea, and apnoea.

Huang et al.’s [29] lymphoid hypertrophy measurements were used for standardisation. In our cohort, adenotonsillar hypertrophy predominated in all groups except 9- to 11-year-old boys, for whom isolated tonsillar hypertrophy was most common. Masoud and Alwadei [30] reported a mean adenoid–nasopharynx ratio of 0.45 in 7- to 11-year-olds, while Tse et al. [39] documented lower adenoid hypertrophy rates (13.9–19.0%) in 12-year-olds in Hong Kong. Growth studies indicate that adenoids reach maximal size by age 5, plateau, then decline around 10–11 years, with a minor increase at 11 years before progressively decreasing [40]. Waldeyer’s ring undergoes characteristic age-related changes in the growing child. Papaioannou et al. [41] found that, in non-snoring children, adenoid and tonsil sizes peak at ages 7–8 and 8–10 years, respectively, before gradually diminishing, while nasopharyngeal and oropharyngeal airway dimensions grow slowly until age 8 and accelerate thereafter. These patterns are consistent with the higher adenotonsillar hypertrophy rates observed in younger subjects in our cohort. In snoring children, lymphoid tissues enlarge early and continue increasing with age; their nasopharyngeal airway grows slowly, while oropharyngeal dimensions stagnate. Thus, adenotonsillar overgrowth narrows the upper airway until 8–10 years in most children. Although adenotonsillar size regresses spontaneously in non-snorers, it persists in snoring children, maintaining a narrowed oropharyngeal lumen. Their nasopharyngeal airway also enlarges with age but remains relatively narrower than in non-SDB peers, sustaining SDB risk.

Adenotonsillar hypertrophy is widely recognised as one of the key risk factors for paediatric OSA [42]. In the current study, a form of lymphoid tissue hypertrophy was present in 92.3% of subjects, comprising combined adenotonsillar hypertrophy (49.5%), isolated tonsillar hypertrophy (36.6%), and isolated adenoid hypertrophy (6.2%). A recent meta-analysis that included thirty-six studies found that children with adenoid and/or tonsillar hypertrophy had significantly greater mandibular plane and articular angles, and smaller SNA and SNB angles [43]. Huang et al. [29] observed that isolated tonsillar hypertrophy in Chinese children correlated with cephalometric measurements of mandibular protrusion, whereas adenoid and adenotonsillar hypertrophy was associated with mandibular retrognathia, and increased sagittal jaw discrepancy and mandibular plane angle. Another large recent study [44] had similar findings—children with isolated adenoid hypertrophy at 9–12 years of age showed increased vertical growth direction, and those with isolated tonsil and combined adenoid and tonsil hypertrophy at 6–12 years of age showed correlations with a longer mandibular body, a more anterior mandible, and a horizontal skeletal Class III pattern. The body of evidence consistently demonstrates correlations between adenotonsillar hypertrophy and craniofacial morphology. A meta-analysis [22] found that the lateral cephalogram provided high diagnostic accuracy (area under the curve: 0.86) for the diagnosis of adenoid hypertrophy and posterior upper airway obstruction. A recent cephalometric study by Zhan et al. [45] found that children with adenoid hypertrophy are independently associated with sagittal skeletal discrepancies and show altered craniofacial growth patterns, and prolonged snoring (>1 year) and oral breathing (>3 months) exacerbate dentofacial protrusion and upper lip (soft tissue) morphological changes. As children with craniofacial imbalances represent a substantial proportion of orthodontic patients, clinician awareness of these associations, and identification of these features via both clinical and radiographic analysis as part of routine clinical examination, is essential for referring patients with a potentially elevated risk for SDB.

In the current study, female subjects exhibited larger airway widths and more superior hyoid positioning than males, with differences becoming more pronounced at 9–10.99 years, likely due to earlier pubertal development in girls. The hyoid bone, which anchors the genioglossus, pharyngeal constrictors, and anterior neck muscles, forms the pharyngeal floor and determines tongue base position. During normal growth, the hyoid descends more in males due to increased mandibular ramus depth and secondary laryngeal descent—changes not seen in females [46]. Inferior hyoid displacement is a consistent finding in both paediatric and adult OSA patients and correlates with disease severity [47]. Posterior displacement of the hyoid and tongue into the hypopharynx further narrows the airway. The lower hyoid position may result from larger tongues in OSA subjects or represent an adaptive response to maintain patency [48]. The present study was limited to static cephalometric measurements and did not assess craniofacial myofunction or dynamic soft tissue behaviour, and therefore cannot identify patients whose SDB aetiology is primarily driven by dysfunction such as tongue base collapse. Nevertheless, the skeletal risk indicators identified remain mechanistically relevant: a retrognathic mandible with backward rotational growth restricts the space available for the tongue, promoting posterior displacement. Combined with palatine tonsillar enlargement, this produces a compounded reduction in oropharyngeal volume that heightens susceptibility to airway obstruction and SDB. The inferior hyoid displacement (increased AH–FH) and reduced minimum posterior airway space (PASmin) observed in our high-angle and Class II subjects are anatomically consistent with this muscular-craniofacial mechanism contributing to SDB risk.

The patient population represented in our study was predominantly Chinese (87.6%). These normative values are thus most applicable to largely Chinese paediatric orthodontic populations in Singapore. In our study, non-Chinese children exhibited a more anteriorly positioned hyoid compared to their Chinese peers, suggesting that ancestral craniofacial differences may be predisposed to distinct SDB risk profiles from an early age. However, because the small number of cephalograms of non-Chinese participants were grouped collectively, inter-ancestral differences could not be analysed in our study comprehensively, and the biological significance of our findings was limited. Ancestral differences in craniofacial and airway dimensions have been reported [27,49]. In Singapore, Tan et al. [50] estimated moderate-to-severe SDB prevalence (AHI 3–15) at 30.5% and sleep apnoea syndrome at 18.1%, with only 9% of affected adults previously diagnosed. Malays (33.8%) and Chinese populations (32.1%) had higher SDB rates than Indians (16.5%) and others (12.0%; *p* = 0.04), despite Chinese populations having the lowest mean BMI. After adjusting for age, sex, and BMI, Indians had lower odds of moderate-to-severe SDB compared to Chinese people (OR 0.82; 95% CI 0.70–0.96; *p* = 0.02). Comparing our findings with those of Masoud et al. [30], who reported normative upper airway cephalometric data in a predominantly White American paediatric cohort of age-matched private orthodontic patients, several points of convergence and divergence are apparent. Nasopharyngeal width was comparable across studies (our Np: 22.65–23.70 mm; Masoud et al. PNS-ad1: 24.00 mm), as was middle airway space (our U-MPW: 8.97–10.44 mm; Masoud et al. MAS: 9.47 mm) and soft palate thickness (our SPT: 7.87–8.60 mm; Masoud et al. UT: 8.84 mm). In contrast, the adenoid-to-nasopharyngeal ratio in our sample (Ad/Np: 0.48–0.55 across age–sex groups) was modestly higher than the A/N ratio reported by Masoud et al., (0.45, SD 0.10), suggesting a relatively greater adenoid burden relative to nasopharyngeal dimensions in our predominantly Chinese sample. Our minimum posterior airway space values (PASmin: 8.80–10.55 mm) were notably smaller than those reported by Masoud et al. (PAS: 11.91 mm); a difference that may reflect genuine population-level variation in oropharyngeal dimensions, differences in how PAS was defined and measured between studies, or both. Hyoid positioning relative to the cervical spine was similarly lower in our sample (AH-CV: 28.04–30.35 mm) compared with Masoud’s H-C3 distance (32.24 mm), though direct equivalence between these landmarks cannot be assumed. Taken together, these comparisons suggest that while mid-pharyngeal airway dimensions are largely concordant between the two populations, adenoid proportionality and posterior airway space may differ meaningfully—findings consistent with the growing body of evidence suggesting ethnic variation in craniofacial and upper airway morphology. These differences underscore the importance of establishing population-specific normative cephalometric reference data for airway assessment. A recent meta-analysis also found white ethnicity to be a protective risk factor for paediatric OSA [42], which is largely consistent with the comparisons between our and Masoud et al.’s [30] study. Comprehensive comparisons of our findings with other groups are limited by the lack of published studies on cephalometric airway measurements of age-matched paediatric orthodontic populations on a global level. Future studies should recruit larger, distinct ancestral cohorts to elucidate specific cephalometric variations.

It is essential to recognise that lateral cephalograms measure only sagittal airway dimensions and omit medio-lateral aspects, neuromuscular tone, and dynamic airway function—all critical contributors to SDB risk [3]. Airway measurements also vary with patient posture and respiratory phase; correlations between upright lateral cephalograms and supine CT scans are weak [23,51,52]. Our study did not examine correlations between cephalometric measurements and SDB symptoms or include sleep monitoring, highlighting opportunities for future research.

Despite these limitations, specific cephalometric features—such as reduced naso- and oropharyngeal airway spaces, mandibular retrognathia, increased total and lower facial height, elevated ANB and mandibular plane angles, lower hyoid position, and narrower inter-canine widths—are consistently reported to be associated with paediatric OSA [53,54,55,56]. Various studies have compared cephalometric airway measurements in patients with and without OSA. A recent meta-analysis including 16 studies found that the parameters of significance (*p* < 0.05) in paediatric OSA with lower heterogeneity for cephalometric measurements were associated with McNamara’s and Linder-Aronson’s analysis, the hyoid bone position, a retrognathic mandible, and an acute cranial base angle [57]. In a large multicentre study assessing children from the United States, South Africa, South Korea, Saudi Arabia, and Japan, Kim et al. [58] found male gender, Middle Eastern ethnicity, body mass index, gonial angle, and inferiorly positioned hyoid to be associated with increased risk of SDB in a paediatric orthodontic population. Su et al. [59] found that in Chinese children aged 2–12 years old, those with OSA had significantly larger adenoid sizes and a larger ratio of the adenoids to the skeletal upper airway width, larger radius of the tonsils, smaller SNA and SNB angles, more inferiorly and posteriorly positioned hyoid bones, a larger thickness of the soft palate (SPT), and smaller inclination angle of the soft palate measured on lateral cephalograms than those of the controls. Xu et al. [32] found that increased A/N ratio, narrowed posterior airway space, decreased SNA and SNB angles, and shortened ramus height measured on cephalograms were observed among OSA children aged 5–10 years. In the 5–7 age group, the A/N ratio and a lower gonial angle explained 40.0% and 14.7% of the variance in the OAHI, respectively. In the 8–10 age group, the BMI z-score and A/N ratio explained 25.2% and 6.6% of the variance in the OAHI, followed by a lower gonial angle and the hyoid-retrognathion distance (19.1% in total). Au et al. [60] similarly reported that Hong Kong children aged 6–11 years with OSA had larger tonsils and more inferior hyoid positions measured on lateral cephalograms than non-OSA children. In Japanese 6–12-year-olds, those with an adenoid/nasopharyngeal width (A/N) ratio ≥ 0.55 had significantly higher mean OAHI (1.8 ± 3.0/h) than those with an A/N < 0.55 (1.0 ± 1.1/h; *p* = 0.048) [61]. Katyal et al. [55] demonstrated statistical support in a systematic review and meta-analysis for an association between craniofacial disharmony and paediatric SDB, and strong support for reduced upper airway width in children with obstructive sleep apnea. Craniofacial morphology and reduced upper airway dimensions are therefore recognised as multifactorial risk factors—rather than independent causal factors—for paediatric SDB, and the normative values reported in the current study are most appropriately used to identify patients whose anatomical profiles overlap with features associated with SDB in the literature.

### 4.1. Limitations

This study has limitations. Although we calibrated radiographs using tracing software to control systematic error, we did not validate 2D cephalometric measurements against 3D imaging in our cohort. Baldini et al. [62] compared 2D and 3D measurements on CBCT and found that midsagittal structures had high agreement, whereas lateral structures showed distortion on 2D images. Jodeh et al. [63] reported significant discrepancies in five of twelve angular measurements between 2D and 3D analyses. Residual postural variability in tongue and soft palate position remains an inherent limitation of static two-dimensional cephalometric imaging. Future studies may incorporate CBCT data or 3D skull models to quantify 2D–3D measurement errors.

This study did not include polysomnographic results, formal otorhinolaryngological evaluation, nor validated sleep questionnaire data. As such, no clinical diagnosis of SDB or OSA can be established in this cohort. The retrospective cross-sectional methodology also precludes longitudinal conclusions regarding the relationship between craniofacial morphology and SDB development. All findings represent cephalometric anatomical associations with established anatomical risk indicators for SDB as described in the literature, and should not be interpreted as clinical causal risk determinations, diagnostic conclusions, or predictive assessments. These represent important limitations on the clinical interpretation of the findings and highlight the need for future interdisciplinary and longitudinal studies incorporating ENT and formal sleep assessment alongside cephalometric data.

This study was conducted in a paediatric orthodontic population attending a national dental centre, which does not represent the general population. Children presenting for orthodontic treatment may differ systematically from the broader population in terms of craniofacial morphology and access to healthcare, potentially introducing selection bias. The normative values reported here should therefore be interpreted within appropriate clinical contexts and should not be generalised to the population cohorts of children.

The study had a high imbalance between Chinese (n = 354, 87.6%) and non-Chinese (n = 50, 12.4%) subgroups, and ethnic heterogeneity within the non-Chinese category, which comprised Malay, Indian, Caucasian, and Eurasian children grouped due to individually small subgroup sizes. While the study adds to the body of research on cephalometric airway measurements in Asia, these factors limit the biological interpretability of between-ancestry comparisons and generalizability to non-Chinese populations. Future studies should recruit larger, adequately powered, ethnically distinct cohorts to enable meaningful inter-ancestry cephalometric comparisons.

This study did not include BMI classification or allergy/medication status in the exclusion criteria. BMI and allergy data were not available in the retrospective clinical records. Both obesity and adenotonsillar hypertrophy, which has close links with allergies and chronic pharyngeal mucosal inflammation, are recognised risk factors for SDB and represent uncontrolled confounders in this analysis. Future prospective studies should incorporate these variables as covariates.

The retrospective design of this study precluded control over precise cephalographic technique and patient positioning, and the absence of prospective data collection limits the ability to establish causal relationships between the craniofacial and airway variables identified. It is also notable that the large number of statistical comparisons conducted increased the risk of Type I errors (false positive findings). However, it was important for comparisons of all measurements to be made. Multiple-testing corrections to reduce Type I errors were not conducted, as these would have increased the risk of Type II errors instead. Readers are advised to interpret individual *p*-values accordingly.

### 4.2. Future Directions

Residual OSA symptoms, such as snoring, can persist after adenotonsillectomy—a primary treatment for paediatric OSA [64]. Future research could identify cephalometric variables predictive of post-surgical outcomes by correlating pre-treatment radiographic measurements with treatment success, incorporate medical history data to compare OSA and non-OSA paediatric orthodontic patients, investigate for cephalometric measures that provide high sensitivity and specificity for identifying SDB, or utilise dynamic imaging (such as cine-MRI) to characterise the four-dimensional relationship between craniofacial hard and soft tissue structures and upper airway function during sleep.

### 4.3. Clinical Implications

A comprehensive paediatric SDB risk assessment protocol in the dental office, involving a combination of medical and dental history reviews, clinical assessment of tonsils, tongue size, and position, the presence of obesity and the patient’s overall growth and development, radiographic evaluations, and an objective risk assessment questionnaire, specifically tailored for children (under 18 years of age)—such as the Paediatric Sleep Questionnaire—is recommended [25]. Orthodontists can use the population-specific normative cephalometric values reported here to contextualise upper airway measurements in paediatric orthodontic patients, especially in Chinese-dominated populations. Patients presenting with a combination of anatomical risk indicators—including male gender, younger age, Class I or II skeletal pattern, elevated mandibular plane angle, reduced posterior airway dimensions, or cephalometric lymphoid tissue hypertrophy—should prompt clinical awareness, and where clinically indicated, referral to a sleep physician or ENT specialist for formal SDB evaluation. Cephalometric assessment is a screening adjunct and does not replace formal clinical sleep assessment via polysomnography. While lateral cephalograms are not recommended for definitive diagnoses of SDB, they may serve as a useful adjunct when clinicians seek to contextualise anatomical findings in patients with clinical concern for SDB.

## 5. Conclusions

This study reports cephalometric norms for upper airway, skeletal, dental, and soft tissue structures, as well as the prevalence of lymphoid hypertrophy, in a 7- to 11-year-old orthodontic population in Singapore. Adenotonsillar hypertrophy was present in nearly half of the subjects, while isolated tonsillar hypertrophy affected about one-third. Younger, male children of Chinese ancestry displayed cephalometric features—including smaller airway dimensions and higher rates of lymphoid tissue hypertrophy—associated with anatomical risk indicators for SDB within the current evidence base linking craniofacial morphology to paediatric SDB. While this data support the potential utility of upper airway cephalometric assessment as a screening adjunct in paediatric orthodontic practice, they represent cross-sectional cephalometric associations and should not be interpreted as causal for SDB, or direct diagnostic predictions.

## Figures and Tables

**Table 1:** Cephalometric measurements.

**Upper Airway**	
**Variables**	**Definitions**
Ad (mm)	Adenoid width; width of adenoid measured on Ba-Ar (perp)
Np (adenoids) (mm)	Nasopharynx width; width of nasopharynx measured on Ba-Ar (perp)
Ad/Np	Ratio of adenoid width to nasopharynx width, to measure adenoid hypertrophy
Tn (mm)	Tonsil width; width of palatine tonsil measured on Go-B
Op (tonsils) (mm)	Oropharyngeal width; width of oropharynx measured on Go-B at the level of the tonsils
Tn/Op	Ratio of palatine tonsil width to oropharynx width, to measure tonsillar hypertrophy
PM-U (mm)	Length of soft palate; distance from PM to U
SPT (mm)	Thickness of soft palate; maximal thickness of the soft palate measured perpendicular to PM-U
NL/PM-U (°)	Inclination of the long axis of the soft palate relative to the nasal line
PM-UPW (mm)	Depth of retropalatal space from PM to UPW, measured parallel to NL
U-MPW (mm)	Depth of the oropharyngeal airway space from U to MPW, measured parallel to FH at the level of the uvula
PASmin (mm)	Shortest distance between the base of the tongue and the posterior pharyngeal wall within the vertical space between the uvula and vallecula, measured parallel to FH
V-LPW (mm)	Depth of the hypopharyngeal airway space from V to LPW, measured parallel to FH
AH-FH (mm)	Vertical position of the hyoid bone, from AH perpendicular to FH
AH-CV (mm)	Horizontal position of the hyoid bone, from AH to CV, on a line parallel to FH
**Skeletal**	
**Variables**	**Definitions**
SNA (°)	The angle between the S-N line and the N-A line
SNB (°)	The angle between the S-N line and the N-B line
ANB (°)	The angle between the N-A line and the N-B line
FMA (°)	Frankfort mandibular plane angle; the angle between the Frankfort horizontal plane and the MdPl
MMPA (°)	Maxillomandibular plane angle; the angle between the MxPl and the MnPl
Sn-MxP (°)	Maxillary plane angle; the angle between the MxPl and the S-N line
LAFH (%)	The ratio of ANS-Me to N-Me
**Dental**	
**Variables**	**Definitions**
U1-SN (°)	The angle between S-N and Ui-Ur lines
U1-MxPl (°)	The angle between MxPl and Ui-Ur lines
L1-MnPl (°)	The angle between MnPl and Li-Lr line
Interincisal angle (°)	The angle between Ui-Ur and Li-Lr lines
Li-APog (mm)	The distance between Li and A-Pog lines
Overjet (mm)	The distance between Ui and Li, parallel to the FH plane
Overbite (mm)	The distance between Ui and Li, perpendicular to the FH plane
**Soft tissue**	
**Variables**	**Definitions**
Ls-E-line (mm)	Distance between Ls and E-line
Li-E-line (mm)	Distance between Li and E-line

**Table 2:** Demographic data of subjects.

Variable		Number of Subjects	% of Subjects
Age and gender			
Male	7- to 8.99-years-old	101	25.0
	9- to 10.99-years-old	102	25.2
Female	7- to 8.99-years-old	101	25.0
	9- to 10.99-years-old	100	24.8
**Antero-posterior skeletal pattern**			
Class I		222	55.0
Class II		45	11.1
Class III		137	33.9
**Vertical skeletal pattern**			
Low-angle		53	13.1
Average-angle		290	71.8
High-angle		61	15.1
**Ancestry**			
Chinese		354	87.6
Non-Chinese	Malay	28	12.4
	Indian	15	
	Caucasian	4	
	Eurasian	3	

**Table 3:** Upper airway measurements grouped by gender and age.

Upper Airway Measurements	Boys		Girls		
	7- to 8.99-Years-Old (N = 101)	9- to 10.99-Years-Old (N = 102)	7- to 8.99-Years-Old (N = 101)	9- to 10.99-Years-Old (N = 100)	*p* Value
Ad (mm)	12.80 (2.88) ^b^	11.45 (2.80) ^a^	11.76 (2.83) ^ab^	11.38 (2.95) ^a^	0.001 *
Np (adenoids) (mm)	23.22 (2.05) ^ab^	23.70 (2.31) ^b^	22.65 (2.17) ^a^	23.35 (2.14) ^ab^	0.007 *
Ad/Np	0.55 (0.12) ^b^	0.48 (0.11) ^a^	0.52 (0.13) ^ab^	0.49 (0.13) ^a^	<0.001 *
Tn (mm)	6.85 (2.38) ^a^	7.25 (2.28) ^a^	7.50 (2.19) ^a^	7.68 (2.24) ^a^	0.057
Op (tonsils) (mm)	10.84 (2.77) ^a^	11.14 (2.97) ^ab^	12.20 (2.84) ^bc^	12.44 (3.32) ^c^	<0.001 *
Tn/Op	0.63 (0.15) ^a^	0.65 (0.13) ^a^	0.62 (0.11) ^a^	0.62 (0.12) ^a^	0.161
PM-U (mm)	26.41 (3.09) ^ab^	27.37 (3.27) ^b^	25.89 (2.72) ^a^	26.51 (2.79) ^ab^	0.005 *
SPT (mm)	8.26 (1.42) ^ab^	8.60 (1.20) ^b^	7.87 (1.14) ^a^	8.13 (1.11) ^a^	<0.001 *
NL/PM-U (°)	127.50 (5.90) ^a^	128.34 (6.16) ^ab^	131.02 (6.09) ^c^	130.13 (6.26) ^bc^	<0.001 *
PM-UPW (mm)	20.27 (3.28) ^a^	21.81 (3.32) ^b^	21.14 (3.30) ^ab^	21.75 (3.46) ^b^	0.003 *
U-MPW (mm)	9.27 (2.58) ^ab^	8.97 (2.31) ^a^	10.01 (2.26) ^bc^	10.44 (2.73) ^c^	<0.001 *
PASmin (mm)	8.89 (3.03) ^ab^	8.80 (2.97) ^a^	10.03 (3.12) ^bc^	10.55 (3.74) ^c^	<0.001 *
V-LPW (mm)	11.49 (3.25) ^a^	12.52 (3.37) ^ab^	13.05 (2.99) ^b^	14.42 (3.52) ^c^	<0.001 *
AH-FH (mm)	69.96 (5.25) ^b^	74.17 (5.62) ^c^	66.75 (4.93) ^a^	71.18 (5.88) ^b^	<0.001 *
AH-CV (mm)	28.04 (3.28) ^a^	28.77 (4.08) ^a^	28.47 (2.98) ^a^	30.35 (3.53) ^b^	<0.001 *

Values are mean (SD). Superscript letters (a–c) denote homogeneous subsets from post-hoc analysis; groups sharing a letter are not significantly different. *p* values are from one-way ANOVA. * *p* < 0.05.

**Table 4:** Overall prevalence of lymphoid tissue morphology.

	Overall (N = 404)	95% Confidence Interval	
		Lower Limit	Upper Limit
Non-hypertrophied lymphoid tissues	31 (7.7%)	5.1%	10.3%
Tonsillar hypertrophy	148 (36.6%)	31.9%	41.3%
Adenoid hypertrophy	25 (6.2%)	3.8%	8.5%
Combined adenotonsillar hypertrophy	200 (49.5%)	44.6%	54.4%

## Data Availability

The de-identified data supporting the conclusions of this article will be made available by the authors on request.

## Supplementary Materials

The following supporting information can be downloaded at: https://www.mdpi.com/article/10.3390/jcm15134991/s1, Table S1: Cephalometric landmarks and reference planes; Table S2: Craniofacial measurements grouped by age and gender; Table S3: Upper airway measurements grouped by age and gender; Table S4: Craniofacial measurements grouped by antero-posterior skeletal patterns; Table S5: Upper airway measurements grouped by antero-posterior skeletal patterns; Table S6: Craniofacial measurements grouped by vertical skeletal patterns; Table S7: Upper airway measurements grouped by vertical skeletal patterns; Table S8: Craniofacial measurements grouped by ancestry; Table S9: Upper airway measurements grouped by ancestry.
