# Supplementary material for: Paediatric Sleep-Disordered Breathing: Pharyngeal Airway and Lymphoid Tissues in Risk Assessment
Source: J Clin Med. 2026 Jun 26;15(13):4991. doi: 10.3390/jcm15134991 (PMC13362586; doi:10.3390/jcm15134991)
Supplement: Supplementary file 1 [file jcm-15-04991-s001.zip › jcm-4223532-supplementary.pdf]

Supplementary materials

Table S1: Cephalometric landmarks and reference planes

Landmarks	
**Variables**	**Definitions**
ANS	Anterior nasal spine, the tip of the median, sharp bony process of the maxilla
PNS	Posterior nasal spine, the tip of the posterior spine of the palatine bone of the hard palate
Ba	Basion, the most inferior-posterior point on the anterior margin of foramen magnum
Ar	Articulare, intersection of inferior contour of the posterior cranial base and posterior contour of the ramus
PM	Pterygo-maxillare, the point at the junction of the pterygo-maxilla and the posterior nasal spine
U	Uvula, the tip of the uvula
V	Vallecula, the intersection of the epiglottis and the base of the tongue
UPW	Upper pharyngeal wall, point of intersection of the line NL to the posterior pharyngeal wall
MPW	Middle pharyngeal wall, intersection of a line parallel to FH from U, and the posterior pharyngeal wall
LPW	Lower pharyngeal wall, intersection of a line parallel to FH from V, and the posterior pharyngeal wall
AH	Anterior hyoid, the most anterior and superior point on the body of the hyoid bone
C2	2nd cervical vertebrae, the point at the most anterior-inferior position on the second cervical vertebrae
C3	3rd cervical vertebrae, the point at the most anterior-inferior position on the third cervical vertebrae
S	Center of the sella turcica

Po	Porion, the mid point of the line connecting the most superior point of the external auditory canal on both sides
Or	Orbitale, the lowest point on the average of the left and right inferior borders of the bony orbit
N	Nasion, the deepest point in the concavity of the nasofrontal suture
A	A point, the deepest point in the concavity of the anterior maxilla between the anterior nasal spine and the alveolar crest
Ui	Upper incisor, incisal edge
Ur	Upper incisor, apex
Li	Lower incisor, incisal edge
Lr	Lower incisor, apex
B	B point, the deepest point in the concavity of the anterior mandible between the alveolar crest and the pogonion
Pog	Pogonion, the most anterior point on the bony chin
Gn	Gnathion, the most anteroinferior point on the bony chin, between the menton and the pogonion
Me	Menton, the most inferior point on the body chin
Go’	Gonion’ point, the intersection of the tangents of inferior and posterior borders of the mandible
Cd	Condylion, the most posterosuperior point of the condylar head
LaS	Labrale superius, the most anterior point on the margin of the upper membranous lip
LaI	Labrale inferius, the most anterior point on the margin of the lower membranous lip
PRN	Pronasale, Most anterior point of the nasal tip
Pog’	Most anterior point on the soft tissue outline of the chin
**Reference planes**	
**Variables**	**Definitions**
FH	Frankfort horizontal plane, line joining Or and Po

NL	Nasal line, line joining the ANS and Pm
MxPl	Maxillary plane, equal to NL
CV	Cervical vertebrae, the line joining the C2 and C3
Ba-Ar	Tangential line to the anterior margin of the basioocciput
Ba-Ar (Perp)	Line perpendicular to Ba-Ar, at the most convex point of the adenoid shadow
Go-B	Line joining Go and B
MnPl	Mandibular plane, line joining Me and Go
E-line	Line joining PRN and Pog'

Table S2: Craniofacial measurements grouped by age and gender

Measurement	Boys		Girls		p value (ANOVA)
	7- to 8.99-years old (N=101)	9- to 10.99-years old (N=102)	7- to 8.99-years old (N=101)	9- to 10.99-years old (N=100)	
**SNA**	80.60 (3.58)[a]	81.17 (3.28)[ab]	81.97 (3.17)[b]	81.71 (3.78)[ab]	0.026*
**SNB (º)**	78.72 (3.76)[ab]	78.41 (3.95)[a]	79.82 (3.30)[b]	79.70 (3.74)[ab]	0.013*
**ANB (º)**	1.88 (2.80)[a]	2.76 (3.03)[a]	2.15 (2.53)[a]	2.01 (3.34)[a]	0.151
**FMA (º)**	27.05 (4.43)[a]	26.53 (5.51)[a]	26.68 (5.08)[a]	26.59 (5.64)[a]	0.893
**MMPA (º)**	26.98 (4.27)[a]	26.47 (4.79)[a]	26.44 (4.98)[a]	25.93 (5.58)[a]	0.519

Measurement	Boys		Girls		p value (ANOVA)
	7- to 8.99-years old (N=101)	9- to 10.99-years old (N=102)	7- to 8.99-years old (N=101)	9- to 10.99-years old (N=100)	
**SN-MXP (º)**	8.78 (3.02)[a]	8.97 (3.03)[a]	8.09 (3.20)[a]	10.57 (17.25)[a]	0.255
**LAFH (%)**	53.99 (1.95)[a]	53.59 (1.83)[a]	54.24 (2.04)[a]	53.85 (2.14)[a]	0.128
**U1 to SN (º)**	103.99 (8.57)[a]	106.40 (10.02)[ab]	105.97 (7.96)[ab]	107.74 (9.39)[b]	0.032*
**U1MxP (º)**	112.77 (8.13)[a]	115.37 (9.45)[ab]	114.06 (8.40)[ab]	116.50 (8.99)[b]	0.017*
**L1 to MdP (º)**	89.33 (6.29)[a]	89.59 (7.24)[a]	90.51 (7.78)[a]	88.82 (8.63)[a]	0.445
**Interincisal angle (º)**	130.92 (10.35)[a]	128.57 (12.22)[a]	128.98 (10.79)[a]	128.74 (12.60)[a]	0.437
**L1i to APg (mm)**	3.66 (2.14)[a]	3.60 (2.31)[a]	3.66 (2.04)[a]	3.77 (2.60)[a]	0.963
**Overjet (mm)**	0.47 (3.12)[a]	2.52 (4.48)[c]	1.03 (2.93)[ab]	2.00 (4.32)[bc]	<0.001*
**Overbite (mm)**	1.56 (2.15)[ab]	2.19 (2.19)[b]	1.01 (1.75)[a]	2.23 (2.29)[b]	<0.001*
**Ls/E-line (mm)**	0.39 (2.45)[a]	0.44 (2.52)[a]	0.22 (2.07)[a]	-0.39 (2.61)[a]	0.059
**Li'/E-line (mm)**	3.26 (2.54)[b]	2.63 (2.36)[ab]	3.00 (2.03)[ab]	2.22 (2.58)[a]	0.013*

Mean(SD) were presented; Different letters indicate statistically significant differences

Measurement	Boys		Girls		p value (ANOVA)
	7- to 8.99-years old (N=101)	9- to 10.99-years old (N=102)	7- to 8.99-years old (N=101)	9- to 10.99-years old (N=100)	
**Overjet (mm)**	0.47 (3.12)[a]	2.52 (4.48)[c]	1.03 (2.93)[ab]	2.00 (4.32)[bc]	<0.001*
**Overbite (mm)**	1.56 (2.15)[ab]	2.19 (2.19)[b]	1.01 (1.75)[a]	2.23 (2.29)[b]	<0.001*

Table S3: Upper airway measurements grouped by age and gender

Measurement	Boys		Girls		p value (ANOVA)
	7- to 8.99-years old (N=101)	9- to 10.99-years old (N=102)	7- to 8.99-years old (N=101)	9- to 10.99-years old (N=100)	
**Airway measurements**					
**Ad (mm)**	12.80 (2.88)[b]	11.45 (2.80)[a]	11.76 (2.83)[ab]	11.38 (2.95)[a]	0.001*
**Np (adenoids) (mm)**	23.22 (2.05)[ab]	23.70 (2.31)[b]	22.65 (2.17)[a]	23.35 (2.14)[ab]	0.007*
**Ad/Np**	0.55 (0.12)[b]	0.48 (0.11)[a]	0.52 (0.13)[ab]	0.49 (0.13)[a]	<0.001*
**Tn (mm)**	6.85 (2.38)[a]	7.25 (2.28)[a]	7.50 (2.19)[a]	7.68 (2.24)[a]	0.057
**Op (tonsils) (mm)**	10.84 (2.77)[a]	11.14 (2.97)[ab]	12.20 (2.84)[bc]	12.44 (3.32)[c]	<0.001*
**Tn/Op**	0.63 (0.15)[a]	0.65 (0.13)[a]	0.62 (0.11)[a]	0.62 (0.12)[a]	0.161
**PM-U (mm)**	26.41 (3.09)[ab]	27.37 (3.27)[b]	25.89 (2.72)[a]	26.51 (2.79)[ab]	0.005*
**SPT (mm)**	8.26 (1.42)[ab]	8.60 (1.20)[b]	7.87 (1.14)[a]	8.13 (1.11)[a]	<0.001*
**NL/PM-U (º)**	127.50 (5.90)[a]	128.34 (6.16)[ab]	131.02 (6.09)[c]	130.13 (6.26)[bc]	<0.001*
**PM-UPW (mm)**	20.27 (3.28)[a]	21.81 (3.32)[b]	21.14 (3.30)[ab]	21.75 (3.46)[b]	0.003*
**U-MPW (mm)**	9.27 (2.58)[ab]	8.97 (2.31)[a]	10.01 (2.26)[bc]	10.44 (2.73)[c]	<0.001*

Measurement	Boys		Girls		p value (ANOVA)
	7- to 8.99-years old (N=101)	9- to 10.99-years old (N=102)	7- to 8.99-years old (N=101)	9- to 10.99-years old (N=100)	
**PASmin (mm)**	8.89 (3.03)[ab]	8.80 (2.97)[a]	10.03 (3.12)[bc]	10.55 (3.74)[c]	<0.001*
**V-LPW (mm)**	11.49 (3.25)[a]	12.52 (3.37)[ab]	13.05 (2.99)[b]	14.42 (3.52)[c]	<0.001*
**AH-FH (mm)**	69.96 (5.25)[b]	74.17 (5.62)[c]	66.75 (4.93)[a]	71.18 (5.88)[b]	<0.001*
**AH-CV (mm)**	28.04 (3.28)[a]	28.77 (4.08)[a]	28.47 (2.98)[a]	30.35 (3.53)[b]	<0.001*

Mean(SD) were presented; Different letters indicate statistically significant differences

Table S4: Craniofacial measurements grouped by antero-posterior skeletal patterns

Measurement	Class I (N=222)	Class II (N=45)	Class III (N=137)	p value (ANOVA)
**Non-airway measurements**				
**SNA (º)**	81.55 (3.26)[b]	83.69 (3.67)[c]	80.30 (3.38)[a]	<0.001*
**SNB (º)**	78.45 (3.18)[b]	76.39 (3.45)[a]	81.23 (3.65)[c]	<0.001*
**ANB (º)**	3.10 (1.36)[b]	7.30 (1.05)[c]	-0.93 (1.59)[a]	<0.001*

Measurement	Class I (N=222)	Class II (N=45)	Class III (N=137)	p value (ANOVA)
**FMA (º)**	27.22 (5.10)[b]	28.34 (5.09)[b]	25.35 (5.05)[a]	<0.001*
**MMPA (º)**	26.80 (4.89)[b]	28.74 (4.22)[c]	25.15 (4.85)[a]	<0.001*
**SN-MXP (º)**	8.88 (2.93)[a]	8.59 (2.98)[a]	9.62 (14.93)[a]	0.691
**LAFH (%)**	53.87 (1.97)[a]	54.19 (2.32)[a]	53.91 (1.95)[a]	0.617
**U1 to SN (º)**	105.06 (8.96)[a]	107.61 (9.46)[a]	107.06 (9.05)[a]	0.058
**U1MxP (º)**	113.94 (8.80)[a]	116.20 (9.31)[a]	115.37 (8.68)[a]	0.154
**L1 to MdP (º)**	90.69 (7.39)[b]	93.80 (7.33)[c]	86.35 (6.57)[a]	<0.001*
**Interincisal angle (º)**	128.57 (11.18)[b]	121.26 (11.28)[a]	133.13 (10.59)[c]	<0.001*
**L1i to APg (mm)**	3.41 (2.32)[b]	2.31 (2.43)[a]	4.54 (1.79)[c]	<0.001*
**Overjet (mm)**	2.01 (3.30)[b]	6.83 (3.66)[c]	-1.05 (2.34)[a]	<0.001*
**Overbite (mm)**	1.36 (1.87)[a]	2.97 (2.11)[c]	1.96 (2.42)[b]	<0.001*
**Ls/E-line (mm)**	0.59 (1.97)[b]	3.05 (2.27)[c]	-1.47 (1.98)[a]	<0.001*
**Li′/E-line (mm)**	2.77 (2.52)[a]	3.52 (2.52)[a]	2.54 (2.13)[a]	0.061

Mean(SD) were presented; Different letters indicate statistically significant differences

Table S5: Upper airway measurements grouped by antero-posterior skeletal patterns

Measurement	Class I (N=222)	Class II (N=45)	Class III (N=137)	p value (ANOVA)
**Airway measurements**				
**Ad (mm)**	12.04 (3.01)[a]	11.79 (2.35)[a]	11.55 (2.92)[a]	0.305
**Np (adenoids) (mm)**	23.05 (2.09)[a]	22.76 (2.08)[a]	23.68 (2.34)[b]	0.009*
**Ad/Np**	0.52 (0.13)[b]	0.52 (0.10)[ab]	0.49 (0.12)[a]	0.037*
**Tn (mm)**	7.16 (2.14)[a]	6.86 (2.00)[a]	7.74 (2.54)[a]	0.022*
**Op (tonsils) (mm)**	11.43 (2.81)[a]	10.91 (2.53)[a]	12.26 (3.46)[b]	0.010*
**Tn/Op**	0.63 (0.13)[a]	0.63 (0.14)[a]	0.64 (0.13)[a]	0.894
**PM-U (mm)**	26.84 (3.07)[b]	27.47 (3.40)[b]	25.76 (2.61)[a]	<0.001*
**SPT (mm)**	8.18 (1.24)[a]	8.09 (1.23)[a]	8.32 (1.26)[a]	0.445
**NL/PM-U (º)**	129.77 (5.98)[b]	132.90 (5.66)[c]	127.19 (6.14)[a]	<0.001*
**PM-UPW (mm)**	21.11 (3.54)[a]	22.25 (2.67)[a]	21.13 (3.31)[a]	0.106
**U-MPW (mm)**	9.56 (2.41)[a]	9.51 (2.42)[a]	9.89 (2.75)[a]	0.441
**PASmin (mm)**	9.48 (3.08)[a]	9.10 (2.99)[a]	9.85 (3.72)[a]	0.346

Measurement	Class I (N=222)	Class II (N=45)	Class III (N=137)	p value (ANOVA)
**V-LPW (mm)**	12.81 (3.33)[a]	12.51 (2.96)[a]	13.06 (3.77)[a]	0.616
**AH-FH (mm)**	70.22 (6.00)[a]	71.52 (6.38)[a]	70.70 (5.98)[a]	0.384
**AH-CV (mm)**	28.84 (3.25)[a]	28.54 (3.63)[a]	29.12 (4.07)[a]	0.603

Mean(SD) were presented; Different letters indicate statistically significant differences

Table S6: Craniofacial measurements grouped by vertical skeletal patterns

Measurement	Low angle (N=53)	Average angle (N=290)	High angle (N=61)	p value (ANOVA)
**Non-airway measurements**				
**SNA (º)**	81.76 (3.39)[a]	81.45 (3.50)[a]	80.61 (3.46)[a]	0.154
**SNB (º)**	81.24 (3.98)[c]	79.18 (3.56)[b]	77.24 (3.33)[a]	<0.001*
**ANB (º)**	0.52 (2.73)[a]	2.26 (2.85)[b]	3.37 (2.99)[c]	<0.001*
**FMA (º)**	19.68 (4.25)[a]	26.70 (3.84)[b]	32.86 (3.25)[c]	<0.001*
**MMPA (º)**	18.41 (2.68)[a]	26.36 (2.81)[b]	33.90 (2.19)[c]	<0.001*
**SN-MXP (º)**	9.31 (2.91)[a]	9.30 (10.47)[a]	7.99 (2.78)[a]	0.580

Measurement	Low angle (N=53)	Average angle (N=290)	High angle (N=61)	p value (ANOVA)
**LAFH (%)**	52.46 (1.80)[a]	53.88 (1.91)[b]	55.37 (1.56)[c]	<0.001*
**U1 to SN (º)**	111.10 (8.81)[b]	105.68 (9.01)[a]	103.21 (8.06)[a]	<0.001*
**U1MxP (º)**	120.40 (8.93)[c]	114.36 (8.58)[b]	111.20 (7.70)[a]	<0.001*
**L1 to MdP (º)**	93.19 (7.83)[c]	89.96 (7.23)[b]	84.54 (6.13)[a]	<0.001*
**Interincisal angle (º)**	127.99 (12.37)[a]	129.32 (11.74)[a]	130.35 (9.63)[a]	0.554
**L1i to APg (mm)**	3.18 (2.61)[a]	3.71 (2.20)[a]	3.91 (2.31)[a]	0.203
**Overjet (mm)**	1.46 (4.45)[a]	1.38 (3.64)[a]	2.16 (4.23)[a]	0.357
**Overbite (mm)**	2.38 (2.38)[a]	1.66 (2.11)[a]	1.58 (2.10)[a]	0.069
**Ls/E-line (mm)**	-0.83 (2.17)[a]	0.22 (2.44)[b]	0.76 (2.41)[b]	0.002*
**Li'/E-line (mm)**	1.64 (2.35)[a]	2.79 (2.34)[b]	3.71 (2.38)[c]	<0.001*

Mean(SD) were presented; Different letters indicate statistically significant differences

Table S7: Upper airway measurements grouped by vertical skeletal patterns

Measurement	Low angle (N=53)	Average angle (N=290)	High angle (N=61)	p value (ANOVA)
**Airway measurements**				
**Ad (mm)**	10.90 (2.73)[a]	11.90 (2.93)[ab]	12.44 (2.82)[b]	0.016*
**Np (adenoids) (mm)**	23.43 (2.36)[a]	23.16 (2.24)[a]	23.40 (1.82)[a]	0.569
**Ad/Np**	0.46 (0.11)[a]	0.52 (0.13)[b]	0.53 (0.12)[b]	0.007*
**Tn (mm)**	7.49 (3.03)[a]	7.26 (2.12)[a]	7.45 (2.34)[a]	0.709
**Op (tonsils) (mm)**	11.88 (4.03)[a]	11.46 (2.87)[a]	12.37 (2.82)[a]	0.089
**Tn/Op**	0.63 (0.14)[a]	0.64 (0.12)[a]	0.61 (0.15)[a]	0.235
**PM-U (mm)**	26.68 (2.52)[a]	26.63 (3.10)[a]	26.00 (2.97)[a]	0.315
**SPT (mm)**	8.15 (1.27)[a]	8.23 (1.29)[a]	8.23 (1.03)[a]	0.920
**NL/PM-U (º)**	127.77 (6.36)[a]	129.15 (6.09)[ab]	130.97 (6.54)[b]	0.021*
**PM-UPW (mm)**	21.93 (3.24)[a]	21.07 (3.38)[a]	21.45 (3.49)[a]	0.210
**U-MPW (mm)**	10.09 (2.95)[a]	9.56 (2.45)[a]	9.84 (2.53)[a]	0.322
**PASmin (mm)**	9.89 (4.06)[a]	9.49 (3.09)[a]	9.61 (3.59)[a]	0.712
**V-LPW (mm)**	13.11 (3.97)[a]	12.80 (3.32)[a]	12.96 (3.58)[a]	0.813

Measurement	Low angle (N=53)	Average angle (N=290)	High angle (N=61)	p value (ANOVA)
**AH-FH (mm)**	70.30 (6.36)[a]	70.34 (5.70)[a]	71.60 (7.18)[a]	0.323
**AH-CV (mm)**	29.92 (3.84)[b]	28.84 (3.52)[ab]	28.31 (3.54)[a]	0.049*

Mean(SD) were presented; Different letters indicate statistically significant differences

Table S8: Craniofacial measurements grouped by ancestry

Measurement	Chinese (N=354)	Non-Chinese (N=50)	p value (ANOVA)
**Non-airway measurements**			
**SNA (º)**	81.23 (3.48)	82.26 (3.42)	0.052
**SNB (º)**	79.20 (3.76)	78.92 (3.60)	0.623
**ANB (º)**	2.04 (2.92)	3.34 (2.91)	0.003*
**FMA (º)**	27.21 (4.95)	23.19 (5.41)	<0.001*
**MMPA (º)**	26.75 (4.88)	24.36 (4.79)	0.001*
**SN-MXP (º)**	9.27 (9.54)	7.92 (3.02)	0.322
**LAFH (%)**	53.92 (1.95)	53.91 (2.35)	0.975

Measurement	Chinese (N=354)	Non-Chinese (N=50)	p value (ANOVA)
**U1 to SN (º)**	105.69 (8.54)	108.37 (12.16)	0.051
**U1MxP (º)**	114.45 (8.35)	116.29 (11.71)	0.169
**L1 to MdP (º)**	89.02 (7.17)	93.46 (8.81)	<0.001*
**Interincisal angle (º)**	129.79 (10.57)	125.89 (16.53)	0.025*
**L1i to APg (mm)**	3.77 (2.19)	2.95 (2.72)	0.017*
**Overjet (mm)**	1.23 (3.62)	3.48 (4.77)	<0.001*
**Overbite (mm)**	1.66 (2.14)	2.37 (2.19)	0.028*
**Ls/E-line (mm)**	0.16 (2.37)	0.19 (2.91)	0.949
**Li'/E-line (mm)**	2.87 (2.29)	2.11 (3.08)	0.037*

Mean(SD) were presented; Different letters indicate statistically significant differences

Table S9: Upper airway measurements grouped by ancestry

Measurement	Chinese (N=354)	Non-Chinese (N=50)	p value (ANOVA)
**Airway measurements**			
**Ad (mm)**	11.80 (2.92)	12.19 (2.84)	0.370

Measurement	Chinese (N=354)	Non-Chinese (N=50)	p value (ANOVA)
**Np (adenoids) (mm)**	23.27 (2.14)	22.92 (2.54)	0.279
**Ad/Np**	0.51 (0.12)	0.54 (0.12)	0.155
**Tn (mm)**	7.37 (2.28)	6.99 (2.33)	0.270
**Op (tonsils) (mm)**	11.66 (3.02)	11.59 (3.26)	0.868
**Tn/Op**	0.64 (0.13)	0.61 (0.13)	0.114
**PM-U (mm)**	26.48 (3.03)	27.02 (2.89)	0.233
**SPT (mm)**	8.20 (1.22)	8.34 (1.41)	0.442
**NL/PM-U (º)**	129.04 (6.18)	130.67 (6.50)	0.085
**PM-UPW (mm)**	21.16 (3.24)	21.82 (4.25)	0.195
**U-MPW (mm)**	9.63 (2.49)	9.96 (2.83)	0.387
**PASmin (mm)**	9.53 (3.24)	9.77 (3.73)	0.636
**V-LPW (mm)**	12.82 (3.41)	13.18 (3.69)	0.495
**AH-FH (mm)**	70.54 (6.00)	70.42 (6.36)	0.900
**AH-CV (mm)**	28.73 (3.46)	30.10 (4.25)	0.011*

**Measurement**	**Chinese (N=354)**	**Non-Chinese (N=50)**	**p value (ANOVA)**

Mean(SD) were presented; Different letters indicate statistically significant differences
